# Comparison of the effects of different rapid maxillary expansion techniques on craniofacial structures: a finite element analysis study

**DOI:** 10.1186/s40510-023-00459-2

**Published:** 2023-03-06

**Authors:** Nihal Kaya, Elif Dilara Seker, Türker Yücesoy

**Affiliations:** 1 Department of Orthodontics, Institute of Health Sciences, Bezmialem Vakif University, Istanbul, Turkey; 2 Department of Orthodontics, Faculty of Dentistry, Bezmialem Vakif University, Istanbul, Turkey; 3 Private Practice, Istanbul, Turkey

**Keywords:** Cortico-puncture, Rapid maxillary expansion, Finite element method, Pterygomaxillary junction separation

## Abstract

**Aim:**

To compare the effects of three different maxillary expansion appliances with five different types of expansion modalities on stress distribution and displacement on the maxilla and its adjacent craniofacial structures using the finite element method (FEM).

**Materials and methods:**

Cone-beam computed tomography data of a patient with maxillary transverse deficiency were rendered into a three-dimensional model of craniomaxillary structures. The expansion appliances included tooth-borne, hybrid, and bone-borne expanders. Five different expansion modalities were applied to each expander [conventional Rapid Maxillary Expansion (RME) (type 1), midpalatal suture cortico-puncture-assisted RME (type 2), LeFort l cortico-puncture-assisted RME (type 3), surgically assisted RME (SARME) without pterygomaxillary junction (PMJ) separation (type 4), and SARME with bilateral PMJ separation (type 5)]. The numerical and visual data were analyzed.

**Results:**

The highest amount of stress accumulation on teeth was found in the tooth-borne and hybrid groups. On the other hand, more stress concentration on the maxilla was observed in the bone-borne group. SARME cuts with PMJ separation increased total movement by reducing the stress on the midpalatal suture in all groups. While types 1, 2, and 3 were similar in terms of the amounts of displacement, types 4 and 5 increased the total amount of displacement in all groups. The total amounts of displacements from the highest value to the lowest value for the anterior and posterior maxilla were in the bone-borne, tooth-borne, and hybrid groups.

**Conclusions:**

SARME cuts were effective in reducing stress on the teeth, but the cortico-puncture application affected neither the stress values on the teeth nor the transverse displacement in the tooth-borne expanders. Surgical procedures such as SARME and corticotomy should be used with bone-borne devices to improve the outcomes of maxillary expansion procedures.

## Introduction

Rapid maxillary expansion (RME), which is used frequently in patients with transverse maxillary deficiency, was introduced by Angell in 1860 [1]. RME creates changes in both the dentoalveolar region and the midpalatal suture, as well as adjacent craniofacial structures.

The midpalatal suture begins to obliterate during the juvenile period, and the ossification of both the midpalatal suture and circummaxillary sutures increases with age [2]. The increase in the degree of suture fusion makes it difficult to expand the maxilla in patients with transverse maxillary deficiency. Furthermore, this clinical challenge for the expansion procedure may also produce undesirable side effects such as expansion failure, alveolar bone dehiscence, buccal crown tipping, root resorption, reduction in buccal bone thickness, marginal bone loss, pain, edema, and gingival recession [3]. To minimize these side effects, the surgically assisted rapid maxillary expansion (SARME) method was introduced, and this method helps overcome the resistance increased by the bony palate and zygomatic buttress in adults. However, whether the separation of the pterygomaxillary disjunction is necessary is a controversial issue in SARME procedures [4]. Furthermore, SARME procedures with or without pterygomaxillary disjunction have several disadvantages such as the necessity of surgical operation, the requirement of general anesthesia, the possibility of damage to the palatine artery or cranial nerves, high cost, the complex treatment process, and surgical morbidity [5]. Lee et al. [6] introduced the mini-screw-assisted rapid palatal expansion (MARPE) method to reduce surgical risks. However, this technique still has limitations in overcoming areas of resistance located in the midface such as the zygomatic buttresses and the pterygoid junction [7]. Cortico-puncture or micro-osteoperforation methods are applied with the MARPE procedure to overcome bone resistance during maxillary expansion [8]. There is minimal evidence about the effects of micro-osteoperforation or cortico-puncture on maxillary expansion in the literature. Although the patient's age is considered the main factor in the preference of the expansion technique to be used, there is also no consensus about which appliance type or surgical technique should be selected for maxillary expansion in young adults to achieve optimal results.

The finite element method (FEM) is a helpful mathematical tool that was developed by Richard Courant in 1943 and is being used in dentistry today [9]. The main advantage of this method is that it allows the prediction of changes in the anatomical structures of patients where clinical measurement is not possible. Findings that are simulated with FEM may offer many advantages for the improvement of clinical protocols. However, no study has compared cortico-puncture-assisted rapid maxillary expansion to different types of techniques using FEM. The aim of this study was to compare the effects of three different maxillary expansion appliances with five different types of expansion modalities on the maxilla and its adjacent craniofacial structures. We tested the null hypothesis that there are no differences among the maxillary expansion methods included in this study.

## Material and methods

The protocol of this study was approved by the Bezmialem Vakif University Local Ethics Committee (Approval Number: 2021/335). Cone-beam computed tomography data (Large V Smart3D CBCT) of a patient were selected from the institution’s database according to the following criteria: age between 18 and 30 years, maxillary transverse deficiency, no orthodontic treatment history, and no fenestration or dehiscence. The exclusion criteria were cleft lip-palate, craniofacial syndrome, missing teeth, abnormal tooth morphology, and prosthetic restoration.

FEM was performed to evaluate the distribution of the forces on the anatomical structures and the effects of this distribution on the surrounding structures during the maxillary expansion procedure. A three-dimensional model of the craniofacial structures was created by FEM to investigate stress distribution and displacement in teeth, bone, sutures, and surrounding craniofacial structures during maxillary expansion. The FEM model based on cone-beam computed tomography (CBCT) images of a 20-year-old male patient was reconstructed with a slice thickness of 0.1 mm. This model consisted of 9,700,000 elements and 2,300,000 nodes on average, to collect more detailed data of the human skull (Table 1). The reconstructed tomography image was transferred to the 3D Slicer software in the DICOM (DCM) format. The images generated by the CBCT data in the DICOM format were sorted from + 300 to + 400 for cancellous bone and from + 500 to + 1900 for cortical bone in Hounsfield values in the 3D Slicer software and converted into a three-dimensional model by segmentation. After processing these sections, the generated model was exported in the STL format. The three-dimensional model was imported into the Altair Evolve software. The linear material properties of the materials including the Young’s modulus and Poisson's ratio were used in the analyses. The material properties of the analyzed model were defined numerically. Reverse engineering and three-dimensional CAD procedures were carried out with the Altair Evolve software, where appropriate cortical bone, spongiose bone, tooth geometry, pdl, and sutures were modeled according to their Young’s modulus and Poisson’s ratio values (Table 2). The structures that constituted the model were considered isotropic linear elastic, given the limitations in representing the bone’s biomechanical behavior, and their values were used for an initial study of cranial stresses. The considered mechanical properties included the Young’s modulus (E) and Poisson’s ratio.

**Table 1:** Quantitative model data for total nodes and elements

Bonded acrylic cap splint	Type 1	Type 2	Type 3	Type 4	Type 5
Total of nodes	2,395,650	2,401,055	2,381,330	2,387,827	2,416,384
Total of elements	9,760,522	9,784,835	9,701,495	9,724,571	9,841,951

Hybrid expander	Type 1	Type 2	Type 3	Type 4	Type 5
Total of nodes	2,374,011	2,379,567	2,359,039	2,366,232	2,394,742
Total of elements	9,669,553	9,694,548	9,607,910	9,633,681	9,750,645

Bone-borne expander	Type 1	Type 2	Type 3	Type 4	Type 5
Total of nodes	2,377,548	2,383,564	2,500,388	2,369,403	2,398,455
Total of elements	9,684,127	9,711,880	10,524,037	9,645,097	9,767,124

**Table 2:** Poisson’s ratio and Young’s modulus of materials

Materials	Young’s modulus (MPa)	Poisson’s ratio
Cancellous bone	1370	0.3
Cortical bone	13,700	0.3
Tooth	20,000	0.3
Suture	0.68	0.45
PDL	0.69	0.49
Titanium	114,000	0.34
Rapid maxilla expander	200,000	0.33
Maxilla skeletal expander	193,000	0.3
Acrylic resin	2000	0.3

All materials in the models were assumed to be homogenous, isotropic and linearly elastic

The titanium mini screws, acrylic, and rapid maxillary expansion screws were also modeled using this program. The matching process between the mesh structures was carried out in the Altair HyperMesh software for providing force transfer between the models. Mathematical models were formed by dividing geometric models into simple and small pieces called meshes. After the modeling process was completed in the Altair Evolve software, the models were mathematically created with the Altair HyperMesh software and made ready for analysis. The data of the forces were obtained from simulations carried out until reaching a bone distance of 5 mm, which is considered the limit to avoid laceration in the midline. A lateral force of 100 N was applied to the teeth, the alveolar process, and/or the palatal bone using three different appliances with the conventional activation protocol two turns per day, providing 0.25 mm of expansion in each activation step. Furthermore, five different types of expansion modalities were applied for each reconstructed maxillary model. In total, fifteen linear static analyses were performed in this study.

**Fig. 1:**
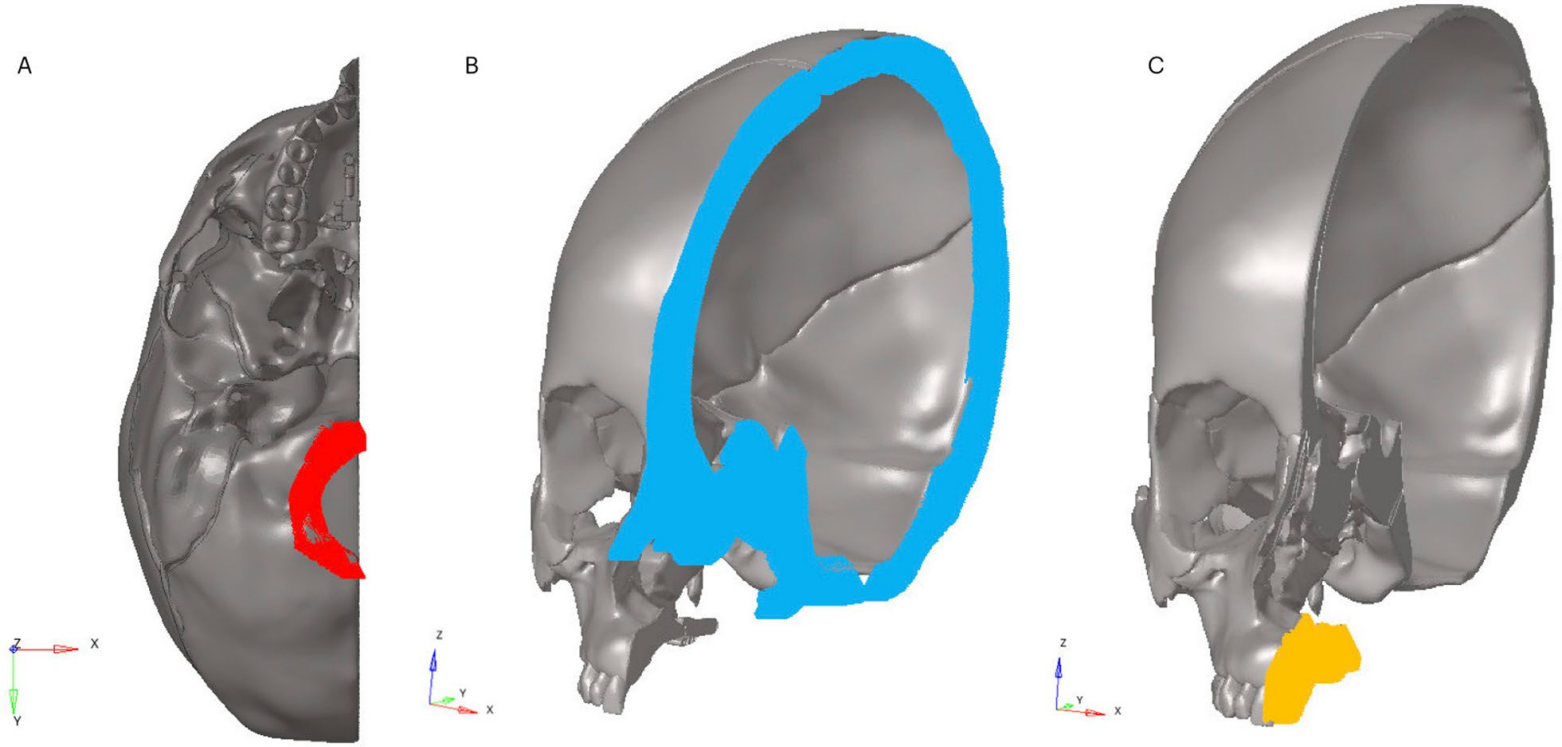
**A** Boundary condition shown in red, **B** Boundary condition shown in blue, **C** Boundary condition shown in orange

The boundary conditions were applied to restrict movements on other planes as shown in Fig. 1 [10].

*Boundary condition in red*: Models were fixed by restricting all degrees of freedom from the nodal points around the foramen magnum to prevent movement in all three axes.

*Boundary condition in blue*: Boundary condition was applied on the *X*-axis normal, symmetrically with respect to the *Y*–*Z* plane.

*Boundary condition in orange*: Boundary condition was applied only to prevent movement in the Y-axis.

The surface relations of the parts forming the model such as bone-suture, bone-periodontal ligament-tooth, bone-appliances, bone-screw, and screw-appliance contact areas were defined by Freeze type contact in the analysis program. This approach was based on the assumption that parts moved with full correlation during their movement.

After all these steps, the models were transferred to the Altair OptiStruct (Altair, Troy, MI, USA) analysis program in the FEM format to perform the analyses.

The arrangement of the three-dimensional mesh structure and its transformation into a mathematically appropriate solid mesh structure, the creation of three-dimensional finite element analysis models, and finite element stress analyses were performed on HP workstations with an Intel Xeon E-2286 processor clocked at 2.40 GHz and 64 GB of ECC memory.

### Designs of the expansion appliances

Bonded acrylic cap splint: McNamara-type (bonded) acrylic cap splint expander with only tooth-borne device with 3 mm of thickness, covering the area from the first premolar to the second molar [11].
Hybrid expander: Both tooth- and bone-borne devices anchored by the first molars and 2 mini screws (SemiOss, Bilimplant, Turkey; diameter: 2 mm; length: 10 mm). Mini screws were placed 2 mm paramedian to the midpalatal suture on the third palatal ruga [12].
(3) Bone-borne expander: Bone-borne device anchored by four mini screws (SemiOss, Bilimplant, Turkey; diameter, 2 mm; length, 10 mm). Two mini screws were placed 2 mm paramedian to the midpalatal suture on the third palatal ruga, and the other two mini screws were placed between the first molars at a distance of 2 mm at the paramedian aspect of the midpalatal suture [12].

**Fig. 2:**
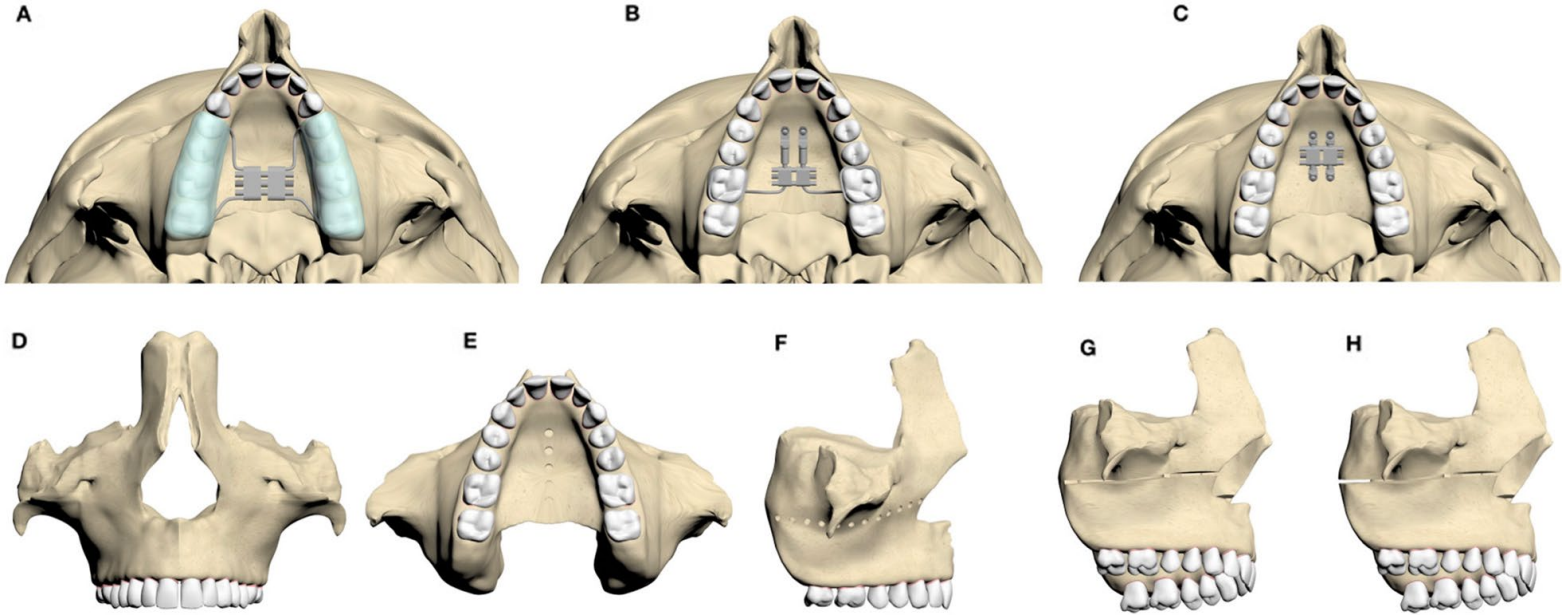
**A** Bonded acrylic cap splint expander, **B** Hybrid expander, **C** Bone-borne expander, **D** Conventional RME protocol, **E** Midpalatal suture cortico-puncture-assisted RME, **F** Lefort l osteotomy line cortico-puncture-assisted RME, **G** SARME without pterygomaxillary separation, **H** SARME with pterygomaxillary separation

The following five types of expansion were applied to each expansion device in the obtained model (Fig. 2).

**Type 1:** Conventional rapid maxillary expansion (RME) protocol.

**Fig. 3:**
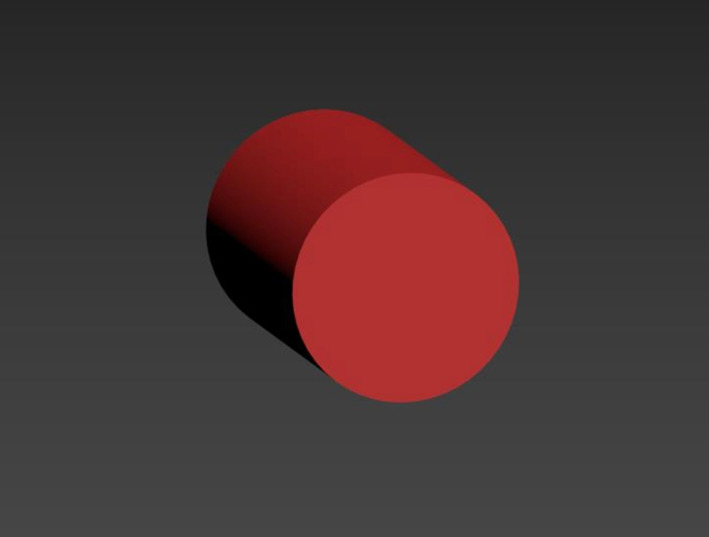
The model created for cortico-puncture application. Trabecular and cortical bone using the subtract method under the Boolean menu removed with a cylinder

**Type 2:** RME assisted with midpalatal cortico-puncture. Cortico-punctures at 1.8 mm of depth from the incisive canal to the mesial of the transverse palatine suture in the palatal bone were created, and the distance between the perforations was 2 mm. While creating the cortico-puncture models, the cylinder with a diameter of 2 mm from the relevant regions was extracted from the trabecular and cortical bone using the subtract method under the Boolean menu (Fig. 3).

**Type 3:** RME assisted with cortico-puncture on the LeFort l osteotomy line. Cortico-punctures at 1.8 mm of depth from the anterior nasal spine to the PMJ on the LeFort l osteotomy line were created, and the distance between the perforations was 2 mm. The cortico-puncture application was performed as described in the type 2 procedure.

**Type 4:** SARME without PMJ separation.

**Fig. 4:**
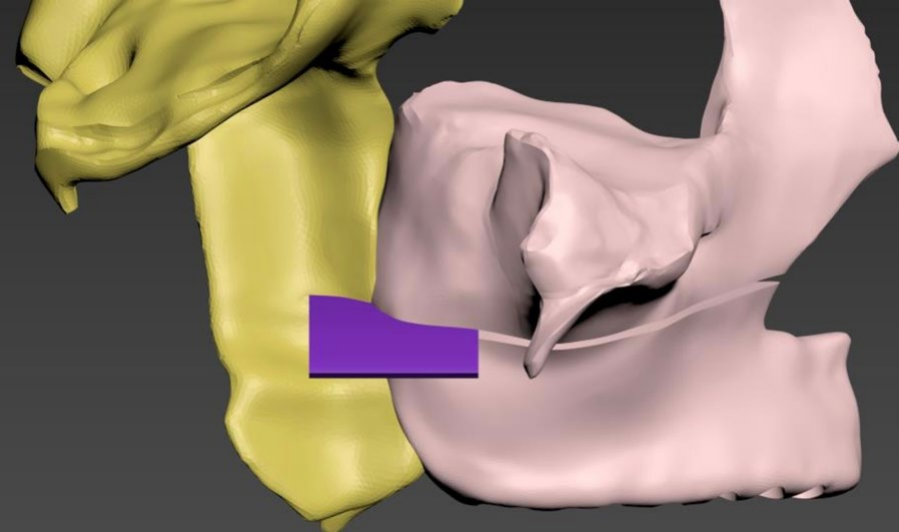
1 mm-thick plane created on the osteotomy line for pterygomaxillary separation

**Type 5:** SARME with bilateral PMJ separation. The separation line (1 mm in thickness) was created by removing the trabecular and cortical bone in the relevant regions using the subtract method under the Boolean menu (Fig. 4).

The von Mises stress values in units of MPa, displacements in millimeters along the X-, Y-, and Z-axes, and total displacement values were analyzed for the selected anatomical structures in all groups.

Positive values on the X-axis represent the lingual direction, negative values represent the buccal direction, positive values on the Y-axis represent the posterior direction, negative values represent the anterior direction, positive values on the Z-axis represent the superior direction, and negative values represent the inferior direction.

## Results

The biomechanical changes obtained with different expansion methods were evaluated under the following main headings.

### Von Mises stress findings

The von Mises stress distribution in the bone and suture structures was analyzed visually using a color map, on which red shows the highest stress concentration, and dark blue shows the lowest stress concentration.

**Fig. 5:**
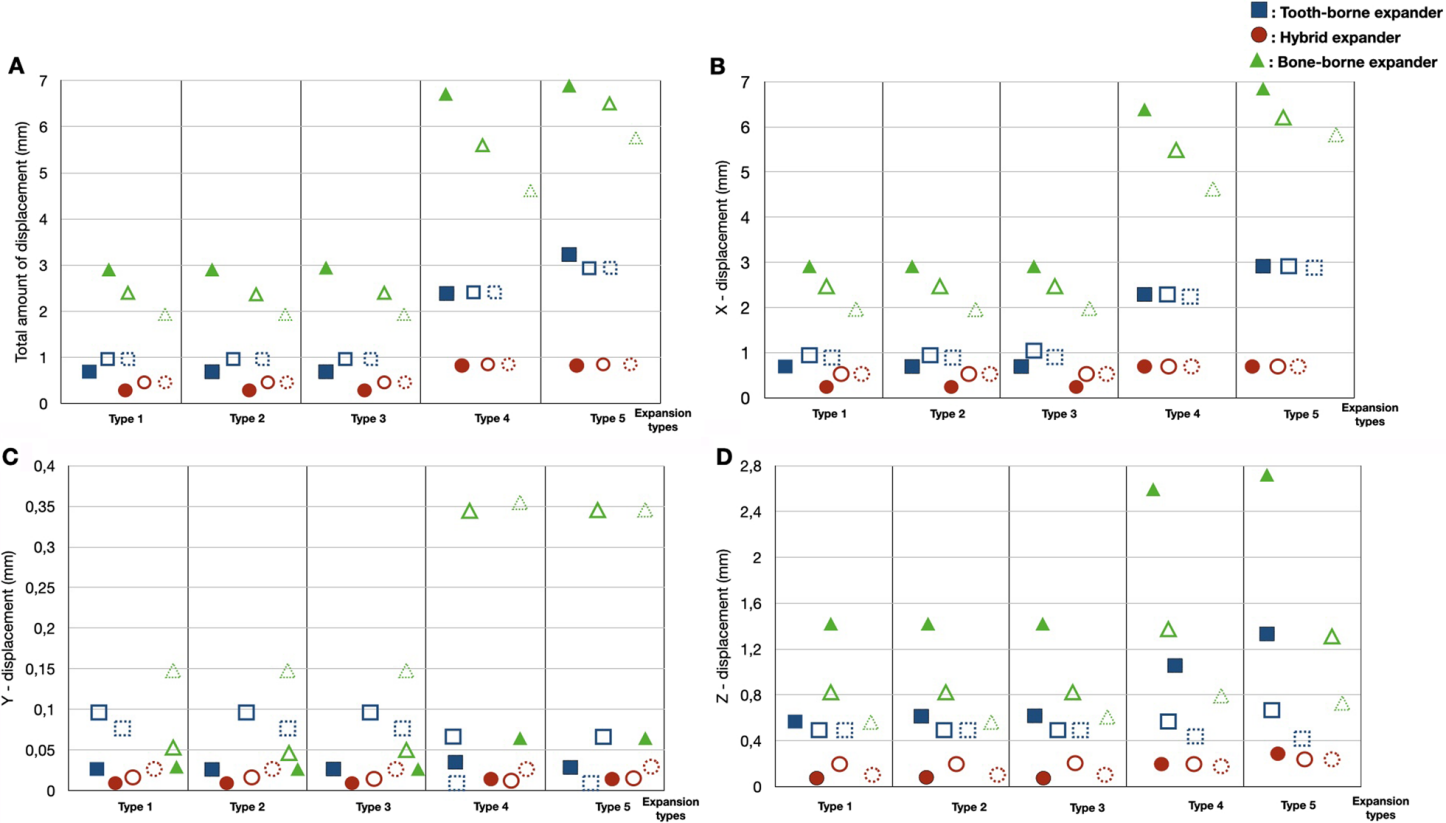
The amount of the displacement for the incisal edge, palatal cusp of the first premolar and palatal cusp of the first molar. **A** Total amount of displacement, **B** Displacements in buccolingual direction, **C** Displacements in anteroposterior direction, **D** Displacements in superoinferior direction. Each group is represented by a different geometric shape. The square shape indicates the tooth-borne group, the rounded shape indicates the hybrid group, and the triangle indicates the bone-borne group. The solid images represent the incisal edge, the unsolid images represent the palatal cusp of the first premolar, and the dotted circle images represent the palatal cusp of the first molar

The stress accumulation degrees ordered from the highest to the lowest among the groups for the maxillary first premolar and first molar teeth were in the tooth-borne, hybrid, and bone-borne groups. There was almost no stress accumulation on the posterior teeth in the bone-borne group. In the tooth-borne group, higher stress values were observed on the posterior teeth for types 1, 2, and 3 among the expansion procedures compared to types 4 and 5. On the other hand, neither LeFort 1 cuts for SARME nor corticotomy procedures were effective in reducing stress accumulation in the hybrid group (Fig. 5).

**Fig. 6:**
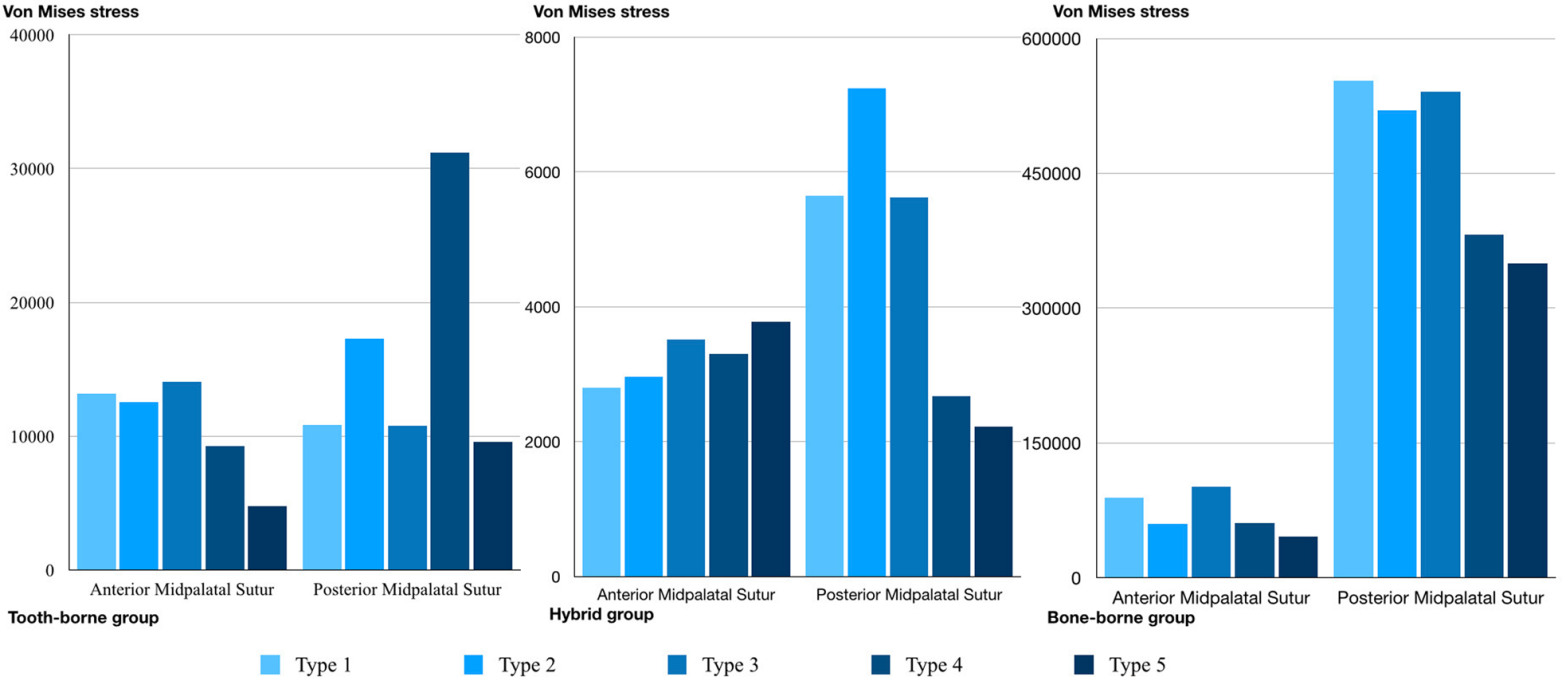
Von Mises stress values of anterior and posterior midpalatal sutures

The greatest stress accumulation on the anterior and posterior midpalatal sutures was in the bone-borne group for all five expansion modalities. While SARME with or without PMJ separation (types 4 and 5) reduced the stress values on the posterior midpalatal suture, similar stress values were observed on the anterior midpalatal suture in the bone-borne group for the five different expansion modalities (Fig. 6). The lowest degree of stress accumulation on the anterior and posterior midpalatal sutures was in the hybrid group for all five expansion modalities. On the other hand, SARME without PMJ separation (type 4) increased the stress values on the posterior midpalatal suture in the tooth-borne group. SARME with PMJ separation (types 4 and 5) reduced the degree of stress accumulation on the posterior midpalatal suture for both the tooth-borne and hybrid groups. While SARME with or without PMJ separation (types 4 and 5) reduced the stress values on the anterior midpalatal suture in the tooth-borne group, similar stress values were observed for the five different expansion modalities on the anterior midpalatal suture in the hybrid group (Fig. 6).

**Fig. 7:**
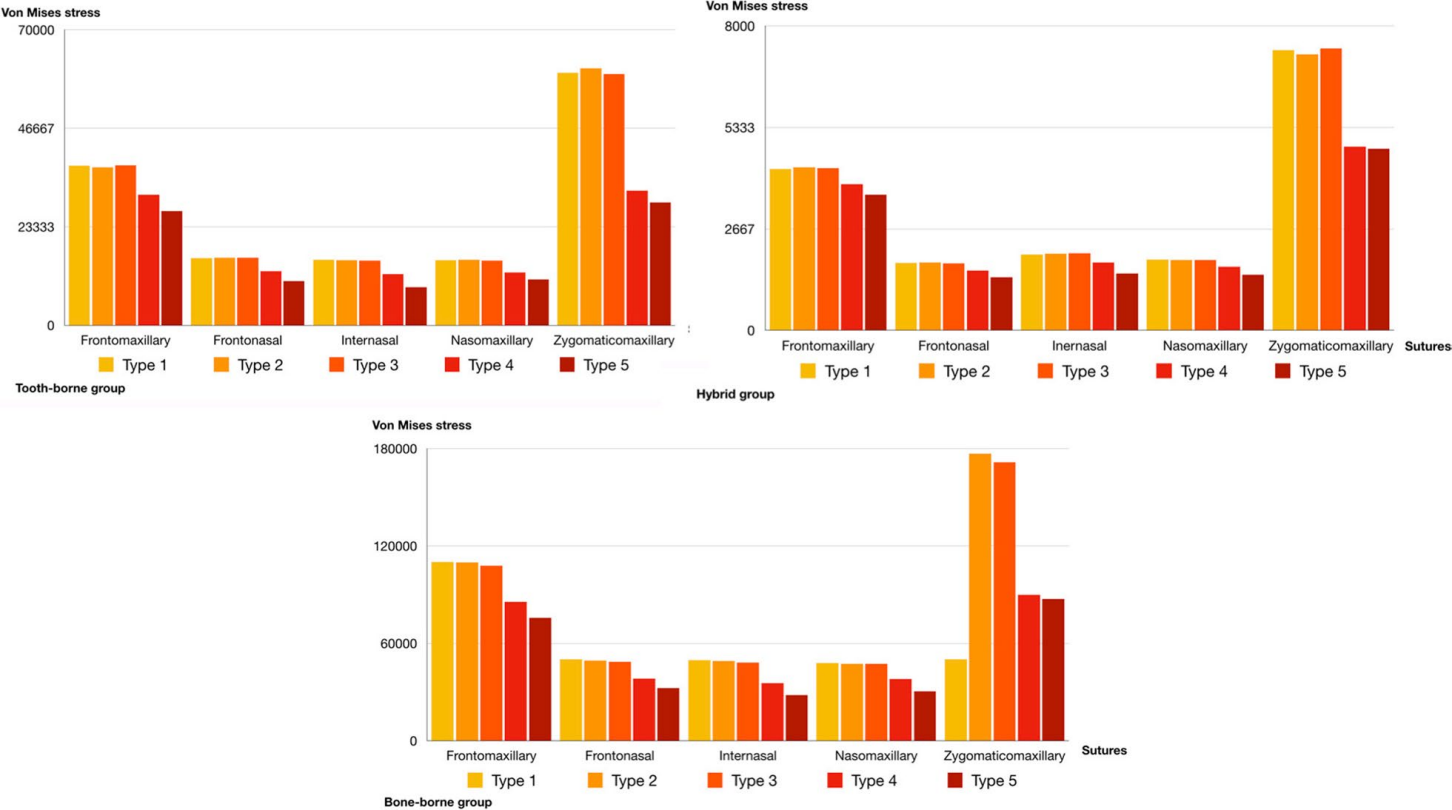
Von Mises stress values of frontomaxillary, frontonasal, internasal, nasomaxillary and zygomaticomaxillary suture

The stress accumulation levels from the highest to the lowest among the groups for the circummaxillary sutures were in the bone-borne, tooth-borne, and hybrid groups. While the greatest stress accumulation among the circummaxillary sutures was on the frontomaxillary and zygomaticomaxillary sutures for all groups, the SARME procedure with or without PMJ separation reduced the stress accumulation values for all sutures in all groups. Furthermore, the cortico-puncture procedures increased the degrees of stress accumulation on the zygomaticomaxillary suture in the bone-borne group (Fig. 7).

### Results on displacement in nasomaxillary complex

The *X*-axis (transverse), *Y*-axis (anteroposterior), *Z*-axis (superoinferior), and total displacement values of the craniofacial structures were analyzed numerically.

### Skeletal findings

**Fig. 8:**
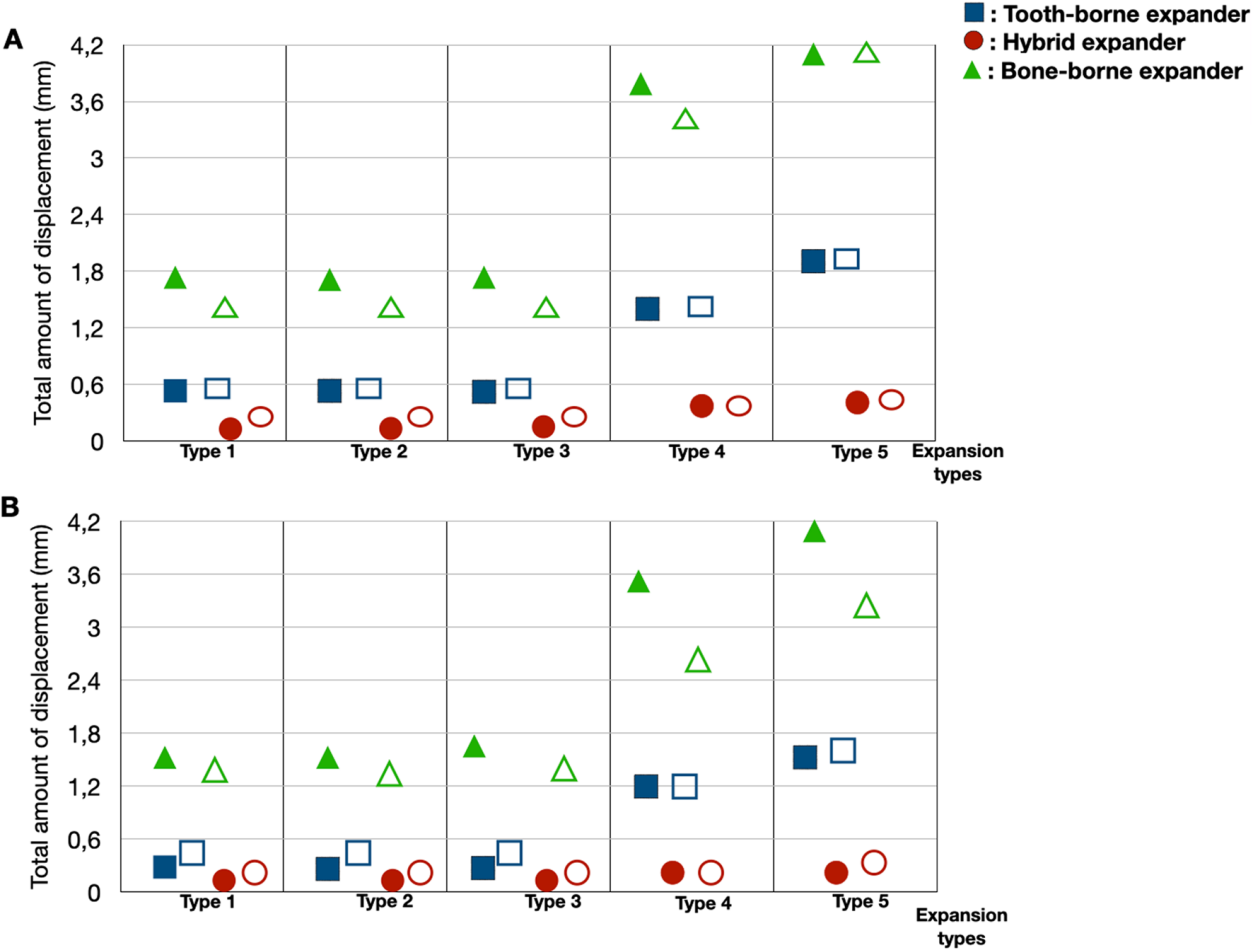
**A** The total amount of displacement of the premolar area and the molar area. The solid images represent the premolar area, and the unsolid images represent the molar area, **B** the total amount of displacement of ANS and PNS points. The solid images represent the ANS point, and the unsolid images represent the PNS points. Each group is represented by a different geometric shape. The square shape indicates the tooth-borne group, the rounded shape indicates the hybrid group, and the triangle indicates the bone-borne group

The total amount of displacements from the highest to the lowest values for the maxillary premolar and molar areas, ANS, and PNS were in the bone-borne, tooth-borne, and hybrid groups. The intragroup comparisons showed that the type 1, 2, and 3 expansion modalities produced similar displacement values, and the type 4 and 5 expansion modalities increased the total amount of displacement in all groups (Fig. 8A, B). Nevertheless, while there was a high similarity in the total amounts of displacements for the premolar and molar areas in the tooth-borne and hybrid groups using the SARME procedure with or without PMJ separation, more displacement was observed with PMJ separation in the molar area compared to the premolar areas in the bone-borne group (Fig. 8).

### Dental findings

#### Total, transverse, and superoinferior displacements

**Fig. 9:**
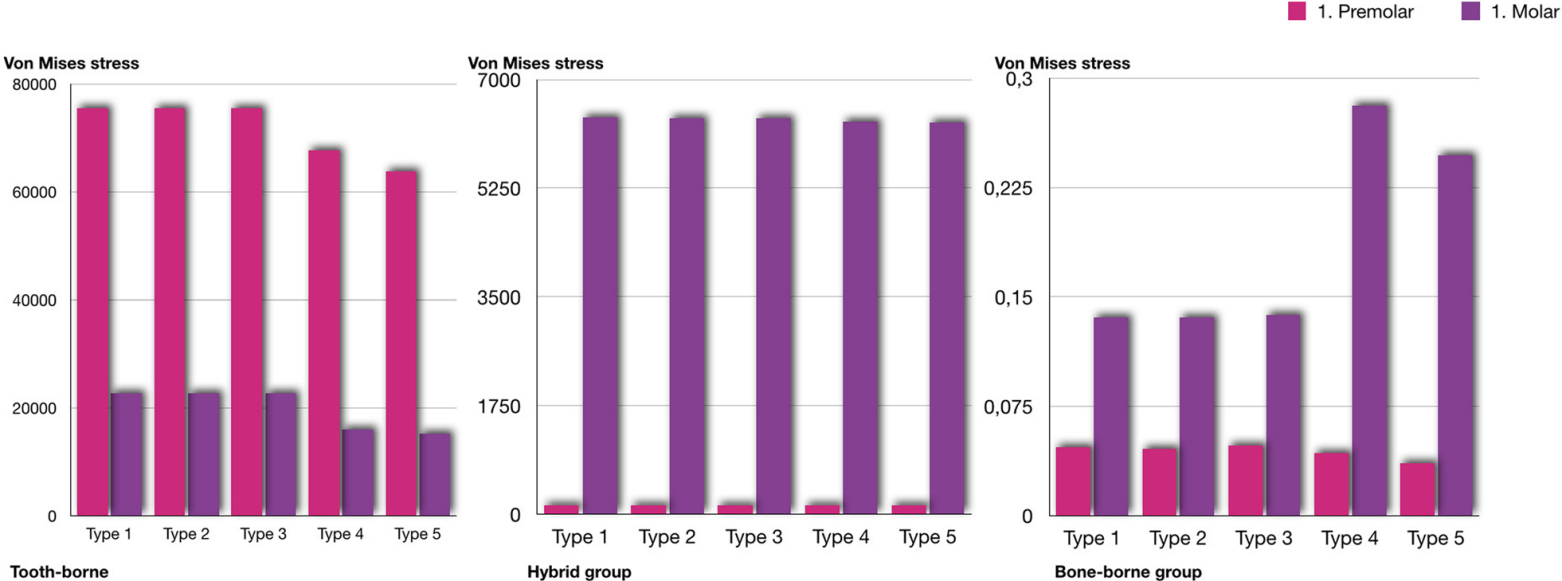
Von Mises stress values of first premolar and first molar

The transverse, superoinferior, and total amounts of displacements from the highest to the lowest values for the incisor, premolar, and molar teeth were in the bone-borne, tooth-borne, and hybrid groups. In the bone-borne group, the highest degree of movement was observed in the incisors, and the lowest degree of movement was in the molar teeth, while the displacements of the teeth were similar among the other groups. Furthermore, the SARME cut did not affect the amount of displacement in the hybrid group, while it increased tooth movement in both the bone-borne and tooth-borne groups. Moreover, the separation of the PMJ increased the movement of the teeth in the transverse direction only in the bone-borne group (Fig. 9).

#### Antero-posterior displacements

The anteroposterior displacement values were very small for all groups. However, an increase in posterior displacement was observed only in the bone-borne group. The separation of the PMJ had no effect in any group in terms of anteroposterior movement.

## Discussion

Maxillary transverse deficiency is a common orthodontic problem, and various expansion methods have been defined for the orthodontic/orthopedic correction of maxillary dentition and basal structures [13]. There is still no consensus on which method will provide optimal expansion for patients with different clinical characteristics such as age and level of bone/suture formation. Although there are many studies investigating maxillary expansion, the orthodontics literature has a lack of evidence in this specific field due to the diversity in factors affecting study designs, such as the appliance type, the expansion protocol, and the patient’s characteristics. This study was conducted to compare the effects of different rapid maxillary expansion methods on craniofacial structures using the CBCT images of the same patient. Due to clinical challenges and ethical issues in comparing many different expansion methods, FEM, which allows the simulation of repeatable biomechanical cases in the craniofacial system and time saving at the same time, was used in this study. Compared to similar studies in the literature, more nodes and elements were used, allowing for a more precise interpretation of the data obtained in this study [14, 15].

The transverse forces delivered during RME initially create compression in the periodontal ligament, bending in the alveolar bone, and tipping in the anchored teeth [16]. Tooth-borne devices have several disadvantages such as buccal tipping or root exposure in the anchor teeth, as well as periodontal problems and the outward rotation of the palatal segments of the maxilla. In this study, the SARME cuts reduced the stress values on the anchored teeth in the tooth-borne group. Nienkemper et al*.* [17] reported that the side effects of RME may be minimized using a hybrid hyrax device that is connected to two orthodontic micro-implants in the anterior palate and attached to the first molars. It was also reported that hybrid devices have significant benefits in terms of less tipping and reduced bone and tooth resorption, especially in the premolar area [18]. Even if the stress values in the hybrid group were lower than those in the tooth-borne group, high levels of stress accumulation were observed on the anchored first molar teeth in the hybrid group, without being affected by the surgical procedures to assist expansion (Fig. 9). This finding suggested that it is more appropriate to select bone-borne devices with four mini screws instead of tooth-borne or hybrid devices regardless of the type of expansion procedure in patients with periodontal problems or thin periodontal biotypes.

Tausche et al*.* [19] reported that a MARPE procedure prevents the buccal tipping of the posterior dentoalveolar segment by 10°. De Oliveria et al*.* [20] stated that a cortico-puncture-assisted hybrid device showed less tipping in anchored teeth. Bud et al*.* [21] found a medium level of molar inclination (2.005°) observed in the midpalatal suture with a cortico-puncture-assisted hybrid device, suggesting that tooth movement cannot be avoided because of the anchorage of the hybrid device at the molar level. In this study, the bone-borne group anchored by four mini screws showed a greater degree of expansion and less tipping of the premolars and molars than those in the hybrid and tooth-borne groups (Fig. 9). However, cortico-puncture applications (types 2 and 3) were not effective in reducing tipping in the premolar and molar teeth in any group. The reason for such differences may be the limited clinical and in vitro studies about this topic and the design differences between studies regarding appliance design and expansion modality.

In this study, the levels stress accumulation and displacement in the midpalatal and circummaxillary sutures were found in descending order as the bone-borne, tooth-borne, and hybrid groups [22, 23]. Although bone anchorage was taken in the hybrid group, the findings of our study showed that better force transfer and displacement were achieved by providing more tooth anchorage in the tooth-borne group. Additionally, the SARME cuts increased the total amount of displacement in all groups. Lucchese et al. reported that LeFort 1 incisions positively affect the movement of the maxilla in the sagittal direction. In this study, we evaluated the effects of LeFort incisions on expansion in the type 4 and type 5 modalities [24]. Seeberger et al*.* [25] reported no significant differences between SARME methods with and without PMJ separation. Our findings were consistent for the tooth-borne and hybrid groups with the study conducted by Seeberger et al*.* Möhlhenrich et al*.* [7] determined in their cadaveric study that SARME without PMJ separation led to a V-shaped transverse maxillary expansion and that SARME with PMJ separation resulted in a parallel transverse expansion in the anterior and posterior regions of the maxilla. In this study, a more parallel displacement between the anterior maxilla and the posterior maxilla was observed in the bone-borne group with the SARME with PMJ separation procedure compared to the SARME without PMJ separation procedure. However, no significant differences were observed in the tooth-borne and hybrid groups (Fig. 8).

Moreover, the tooth-borne and hybrid groups exhibited much lower stress values compared to the bone-borne group, despite the corticotomy and SARME cuts. This finding can be explained by the fact that the expansion force is transmitted to the maxillary teeth at first, and after this, it is indirectly transmitted to the bone structures with tooth-borne appliances. The palatal tissue and anchored teeth absorb the lateral forces applied by tooth-borne and hybrid appliances and transmit less force, causing less stress accumulation on the surrounding structures.

The regions of increased resistance in the facial skeleton due to maxillary expansion are not only the midpalatal suture but also the zygomaxillary buttress and circummaxillary sutures [26]. The findings of this study showed that the stress in the midpalatal suture and the circummaxillary suture was affected by appliance type under similar circumstances. Compared to the degree of stress accumulation in the circummaxillary sutures, the degree of stress accumulation in the midpalatal suture was higher in all expansion modalities in this study. This finding may be explained by the fact that the midpalatal suture is the suture closest to the transverse forces that is produced by the activation of expansion appliances.

Additionally, there were significant differences for all expansion modalities in terms of the amounts of stress accumulation between the anterior part and the posterior part of the midpalatal suture in the bone-borne group compared to the other groups. The midpalatal suture ossification process proceeds from the posterior part of the suture to its anterior part [2]. Therefore, this difference in the bone-borne group can be explained by the fact that transverse forces are transmitted directly to the basal bone, overcoming the resistance of the anterior midpalatal suture, and resulting in higher stress accumulation in the posterior midpalatal and circummaxillary sutures (Figs. 6, 7).

The SARME procedure with PMJ separation significantly reduced the resistance of the posterior midpalatal suture and especially the zygomaticomaxillary suture in the circummaxillary sutures in all groups. Interestingly, the midpalatal cortico-puncture procedure increased the amount of the force transmitted to the posterior midpalatal suture in the tooth-borne and hybrid groups. The cortico-puncture-assisted rapid maxillary expansion procedure, which is considered a current method, has been used in orthodontic patients to avoid the side effects of the SARME protocol in young adults. Bud et al*.* [27] reported that cortico-puncture allowed suture split and accelerated bone remodeling in MARPE in contrast to conventional expansion. However, there is a lack of evidence-based data on cortico-puncture-assisted rapid maxillary expansion on the cranium and surrounding circummaxillary sutures in the literature. This is the first study to investigate the effects of cortico-puncture-assisted rapid maxillary expansion with different expansion modalities using FEM. Additionally, the STL model in this study was generated with 0.1 mm of slice thickness, allowing for more accuracy compared to previous studies [22, 28].

The bone-borne group with cortico-puncture applications (types 2 and 3) showed higher stress accumulation on the zygomaticomaxillary suture, unlike the tooth-borne and hybrid groups. Since cortico-puncture applications do not include the bone cuts used in the SARME procedure, they may have transmitted a greater amount of force to the zygomaticomaxillary suture due to the decrease in the resistance in the midpalatal suture. It was observed that the cortico-puncture applications had an influence on stress accumulation for only the zygomaticomaxillary suture among the circummaxillary sutures in the bone-borne group. A possible explanation for this is that the zygomatic-maxillary suture is close to the center of the applied expansion force, and its location is in parallel with the transverse movement of the maxilla (Fig. 7).

### Limitations

While transferring information from FEM analysis to clinical application, parameters such as methodological differences, the characteristics of the patient, patient age, the position of the mini screws, sex, applied force, appliance design, midpalatal suture maturation degree, surgical modalities, shape of palate, and mini screw position should be carefully evaluated. FEM studies are unable to reflect all clinical conditions such as ethnic differences, the regional acceleration phenomenon (RAP), surrounding musculature system, bone density and variety in sutural maturation degree at the same age, bone metabolism differences, or genetic disorders. These factors may be considered the clinical limitations of this study. However, clinical conditions with multiple different expansion modalities are difficult to apply and standardize on a single patient with the same clinical characteristics as the patient whose data were included in this study. This study will be useful in terms of providing foresight to clinicians and helping future studies.

## Conclusion

In conclusion, the tooth-borne appliances induced a high amount of stress on the teeth, and the stresses on the teeth decreased proportionally with an increase in the amount of bone support. Although higher stress values were observed on the anchor teeth in the tooth-borne appliances compared to the hybrid design, the amount of force localized on the midpalatal and circummaxillary sutures was lower in the hybrid group. It can be assumed that increasing the number of anchor teeth will prevent side effects during maxillary expansion in the anchorage design combining two mini screws and molar teeth. Midpalatal cortico-puncture increased the amount of stress accumulated in the posterior midpalatal area in the tooth-borne and hybrid groups. In contrast, cortico-puncture was not effective in increasing the amount of transverse displacement and vertical control. The highest stress was localized at the zygomaticomaxillary suture among the circummaxillary sutures for all groups, and SARME incision, with or without PMJ separation, reduced the stress accumulated on the sutures and increased transverse displacement.

## Data Availability

The datasets used and/or analyzed during the current study are available from the corresponding author on reasonable request.
